# Oblique Electrostatic Inkjet-Deposited TiO_2_ Electron Transport Layers for Efficient Planar Perovskite Solar Cells

**DOI:** 10.1038/s41598-019-56164-w

**Published:** 2019-12-20

**Authors:** Md. Shahiduzzaman, Toshiharu Sakuma, Tetsuya Kaneko, Koji Tomita, Masao Isomura, Tetsuya Taima, Shinjiro Umezu, Satoru Iwamori

**Affiliations:** 1 Nanomaterials Research Institute (NanoMaRi), Kanazawa University, Kakuma, Kanazawa 920-1192 Japan; 2 Research Institute of Science and Technology (RIST), Tokai University, Kitakaname, Hiratsuka 259-1292 Japan; 3 Department of Chemistry, School of Science, Tokai University, Kitakaname, Hiratsuka 259-1292 Japan; 4 Graduate School of Engineering, Tokai University, Kitakaname, Hiratsuka 259-1292 Japan; 5 Department of Modern Mechanical Engineering, Waseda University, 3-4-1 Ookubo, Shinjuku-ku, Tokyo 269-8555 Japan

**Keywords:** Energy, Solar cells

## Abstract

In this study, a new, simple, and novel oblique electrostatic inkjet (OEI) technique is developed to deposit a titanium oxide (TiO_2_) compact layer (CL) on fluorine-doped tin oxide (FTO) substrate without the need for a vacuum environment for the first time. The TiO_2_ is used as electron transport layers (ETL) in planar perovskite solar cells (PSCs). This bottom-up OEI technique enables the control of the surface morphology and thickness of the TiO_2_ CL by simply manipulating the coating time. The OEI-fabricated TiO_2_ is characterized tested and the results are compared with that of TiO_2_ CLs produced by spin-coating and spray pyrolysis. The OEI-deposited TiO_2_ CL exhibits satisfactory surface coverage and smooth morphology, conducive for the ETLs in PSCs. The power-conversion efficiencies of PSCs with OEI-deposited TiO_2_ CL as the ETL were as high as 13.19%. Therefore, the present study provides an important advance in the effort to develop simple, low-cost, and easily scaled-up techniques. OEI may be a new candidate for depositing TiO_2_ CL ETLs for highly efficient planar PSCs, thus potentially contributing to future mass production.

## Introduction

Within ten years, hybrid organic–inorganic halide perovskite solar cells (PSCs) have become a feasible competitor to conventional silicon-based photovoltaics^1^. Perovskites being used as an active light absorber layer was first reported with power-conversion efficiency (PCE) of 3.8% by Miyasaka *et al*. in 2009^2^, and the best-performing PSC found to date has a remarkable PCE of 25.2%^1^. The electron transport layer (ETL) plays a key role in PSCs because it eliminates the electrical shunt between the transparent electrode/perovskite and transparent electrode/hole-transport layer interfaces, thus enabling and blocking the transport of electrons and blocking holes to the electrodes, respectively^3,4^. Titanium oxide (TiO_2_) is the most frequently used n-type ETL material in PSCs owing to its environmentally friendly nature, tunable electronic properties, low-cost, and consistent energy band alignment with perovskite^5,6^. Therefore, it is of critical importance to produce a uniform, pinhole-free, and full surface coverage TiO_2_ compact layer (CL) as the ETL to realize more efficient electron transport, charge extraction, and low interfacial recombination, which facilitate enhancement in the photovoltaic performance^7^. Moreover, the TiO_2_ CL thickness should be optimized to transport more electrons from perovskite to the fluorine-doped tin oxide (FTO) electrode. Thick TiO_2_ CLs lead to an increase in the distance electrons transport from perovskite to the FTO, which decreases the charge transport^8^. In contrast, a thin TiO_2_ CL cannot efficiently cover the FTO substrate. Thus, TiO_2_ CL thickness optimization has been reported for several deposition techniques including spin-coating (SC)^9,10^, spray-pyrolysis (SP)^11^, atomic layer deposition (ALD)^12^, thermal oxidation^13^, sputtering^14^, chemical vapor deposition (CVD)^15^, inkjet-printing^16^, chemical bath deposition (CBD)^17,18^, electrodeposition (ED)^19^, and so on.

The commonly used SC technique produces low-quality TiO_2_ CLs and is limited in large-scale production. In addition, SP uses an atomizer to spray a titanium precursor onto a heated substrate; the precursor droplets thermally decompose simultaneously to form the TiO_2_ CL^20,21^. High-quality ETLs can be produced through either SC or SP top-down techniques, but these are very sensitive to the control parameters, and hence the PCE of such fabricated PSCs may differ significantly, even for TiO_2_ CLs made by the same procedure. In addition, sputtering^22^ and CVD^23^ require a vacuum environment and have a slow deposition rate, which present challenges for producing TiO_2_ thin films. Scalable bottom-up ALD^24^ requires a relatively long time and is rather costly for preparing TiO_2_ thin films. In addition, although CBD^25^ and ED^26^ enable TiO_2_ layers to be produced at low temperature, it is quite difficult to control the morphology and thickness of the TiO_2_ CL, thus limiting its reproducibility. Therefore, fabricating high-quality, reproducible TiO_2_ CLs for the efficient performance of planar PSCs via a scalable, controllable, and cost-effective technique remains a significant challenge.

Electrostatic inkjet deposition (EI) is a bottom-up strategy that produces TiO_2_ film by discharging in the form of a spray via electrostatic force. In comparison to other bottom-up techniques, EI offers a cost-effective, simple, and promising way to obtain high-quality TiO_2_ film with an easy-to-control thickness as well as large area, multi-stack thin films with high reproducibility. Previously, Umezu *et al*.^27^ reported efficient dye-sensitized solar cells (DSSC) with a PCE of 6.6% by using EI-deposited thicker TiO_2_ thin films. Conventional EI suffers from large droplets that are stacked on the FTO substrate due to gravity. To remove the large droplets, this work modifies conventional EI such that thinner TiO_2_ film can be patterned by changing the substrate position (an ejection angle of 45° with respect to the substrate), which is known as oblique electrostatic inkjet (OEI) deposition. Although it may be implied that OEI enables the fabrication of a smooth surface morphology while allowing for easy control of the TiO_2_ CL thickness, both of which are required for enhancing the performance of PSCs, it remains a challenge to use OEI-deposited TiO_2_ CLs in planar PSCs to achieve good electron injection and extraction.

In this study, we report for the first time a high quality and easily controllable OEI technique that enables patterning of a TiO_2_ CL on the FTO substrate (without the need for a vacuum environment) for efficient planar PSCs. The morphology and optimum thickness of TiO_2_ CL can be controlled in this bottom-up technique by simply manipulating the coating time. Herein, we test and compare the effectiveness of SC-, SP-, and OEI-deposited TiO_2_ CLs s ETLs in PSCs.

## Results and Discussion

**Figure 1:**
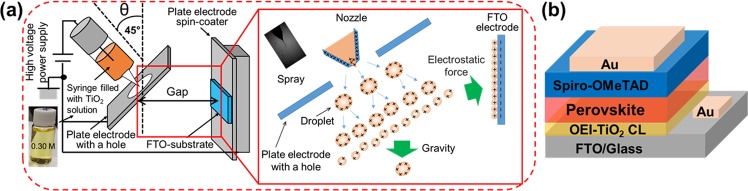
Schematic illustration of (**a**) the OEI setup used to pattern the TiO_2_ CL on FTO glass substrates and (**b**) the device structure of OEI-TiO_2_ CL-based PSCs.

Higher potential TiO_2_ paste was patterned for use in a DSSC by using conventional EI, as described by Umezu *et al*.^27^ This technique has proven to be a very promising technology for printing at high resolution and its capability to eject TiO_2_ from highly viscous liquid. Conventionally, EI discharges TiO_2_ paste in the form of a spray via electrostatic force in the direction perpendicular to the FTO substrate, resulting in large droplets stacked among the thick layers on the resultant substrate due to the gravity acting upon the droplet. The creation and presence of such droplets present limitations in conventional EI to produce thinner and smooth TiO_2_ film for the next generation of PSCs. To overcome this, we modify conventional EI by changing the substrate position to 45° with respect to the substrate, which results in the deposition of high quality TiO_2_ CLs whose thickness is easily controlled. An ejection angle of 45° with respect to the substrate was optimized for smooth morphology and full surface coverage. The CLs prepared by OEI are implemented as ETLs in planar PSCs. Figure 1a shows the schematic illustration of the OEI experimental setup for pattering TiO_2_ CLs. On the lower left, inset a photograph of the precursor shows it is a transparent yellow-orange TiO_2_ solution (concentration of 0.30 M), and the photograph in the upper center of the illustration shows the drop ejection of the spray mode. Overall, one can see the TiO_2_ layer deposition mechanism based on the OEI setup and phenomena. The deposition of large droplets is avoided as these are removed by gravity due to the angle of the spray nozzle. A smooth TiO_2_ CL was deposited based on the electrostatic force. The OEI technique produces TiO_2_ CLs with a smooth morphology and allows the thickness to be controlled by simply optimizing the coating time. Figure 1b reveals a schematic illustration of the device configuration of OEI-TiO_2_ CL-based PSCs.

**Figure 2:**
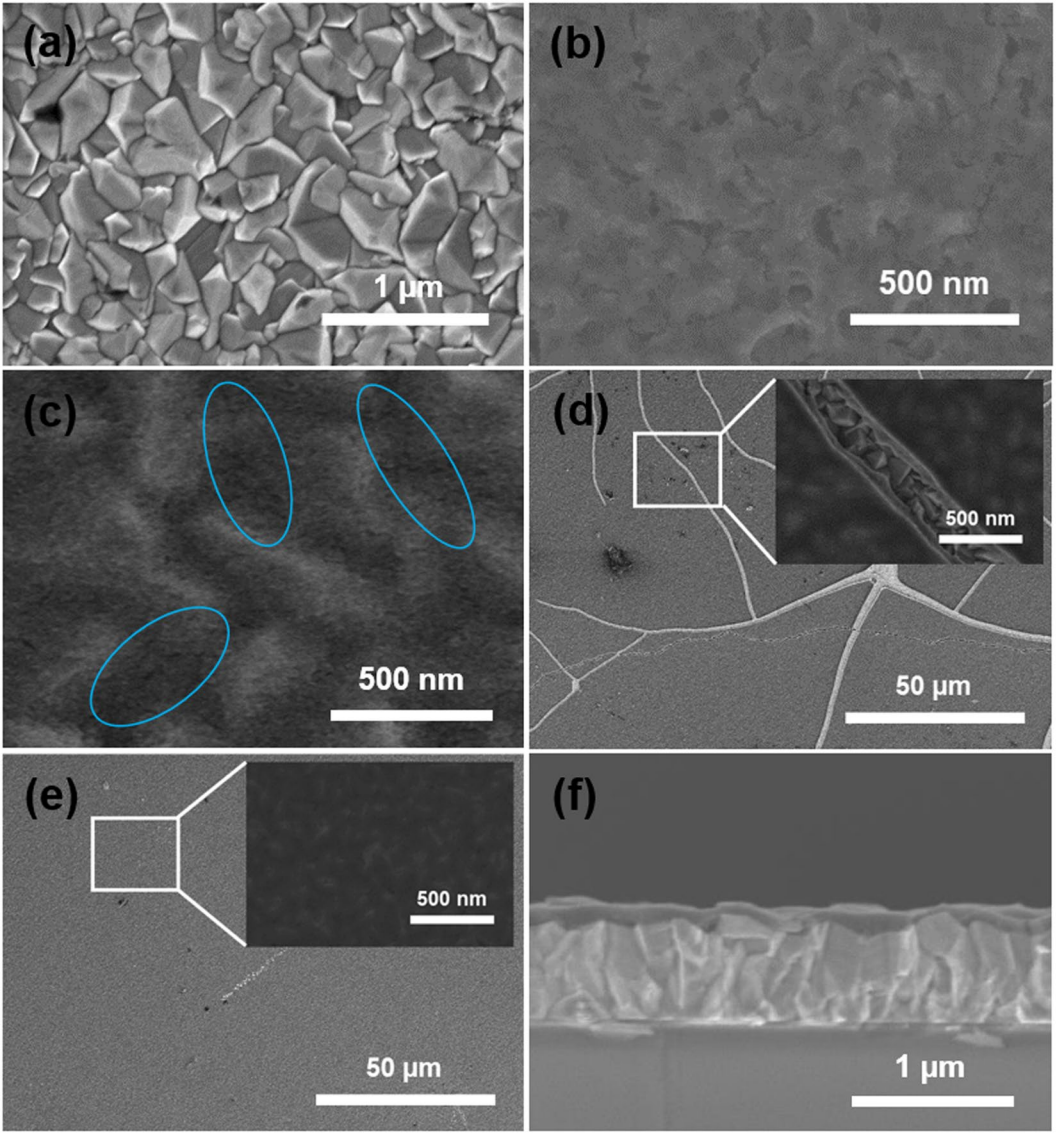
Top-view SEM images of (**a**) bare FTO, (**b**) SC-TiO_2_ CL, (**c**) SP-TiO_2_ CL, (**d**) OEI-TiO_2_ CL-60 sec, and (**e**) OEI-TiO_2_ CL-30 + 30 sec. (**f**) Cross-section SEM image of OEI-TiO_2_ CL-30 + 30 sec film.

Top-view scanning electron microscope (SEM) images of bare FTO and samples SC-TiO_2_ CL, SP-TiO_2_ CL, OEI-TiO_2_ CL-60 sec, and OEI-TiO_2_ CL-30 + 30 sec are shown in Fig. 2a–e, respectively. The bare FTO grains show are rough and range from tens to hundreds of nanometers in size (Fig. 2a). From Fig. 2b, it can be seen that SC-deposited TiO_2_ CL (60 nm thickness) covers smoothly the entire FTO substrate. In addition, as shown in Fig. 2c, the TiO_2_ CL (Optimum thickness of 70 nm) deposited by using SP- holds few cracks (indicated by blue circles), which correlates to the surface of FTO-substrate, followed by linking between FTO and perovskite and facilitates to crucial charge recombination. As shown in Fig. 2d, the OEI-deposited TiO_2_ CL feature a denser smooth scaffold for 60 sec (referred as OEI-TiO_2_ CL-60 sec). The optimized thickness of the OEI-TiO_2_ CL was 100 nm. Some cracks (specified by the white rectangle) occur in the OEI-TiO_2_ CL-60 sec, which may correlate to the FTO grain boundaries, providing possible links between the FTO and perovskite and facilitating extensive charge recombination. To overcome such cracks, the coating time of the OEI-deposited TiO_2_ CL is changed (referred as OEI-TiO_2_ CL-30 + 30 sec), resulting in a uniform, dense scaffold that satisfactorily covers the surface and contains no visible cracks (Fig. 2e). The smooth OEI-TiO_2_ CL-30 + 30 sec samples offer more efficient charge separation and recombination rates, owing to the smooth and entirely crack-free surface coverage. This newly developed OEI technique enables precise control of the morphology, thickness, and deposition rate of the materials for patterning TiO_2_ CLs without a vacuum environment. A cross-sectional SEM image of the OEI-TiO_2_ CL-30 + 30 sec film is shown in Fig. 2f, which was used to measure the TiO_2_ CL thickness.

**Figure 3:**
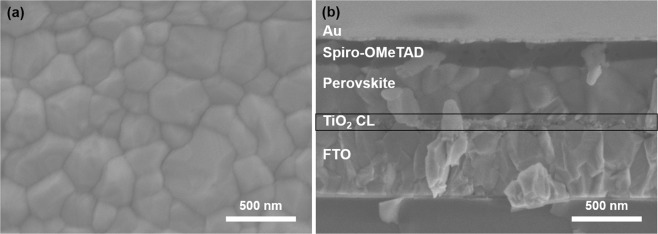
SEM image of the perovskite film grown on (**a**) OEI-TiO_2_ CL-30 + 30 sec and (**b**) cross-sectional SEM image of the planar PSC processed with OEI-TiO_2_ CL-30 + 30 sec.

The surface morphology of perovskite on the OEI TiO_2_ CL is shown in Fig. 3a. The homogeneous surface morphology with large grains induce fewer grain boundaries and thus fewer traps and charge carrier losses. Generally, trap states generated at grain boundaries should be minimized for efficient PSC operation^28^. A cross-sectional SEM image of a complete PSC device made with the OEI-TiO_2_ CL-30 + 30 sec sample as the ETL is shown in Fig. 3b.

**Figure 4:**
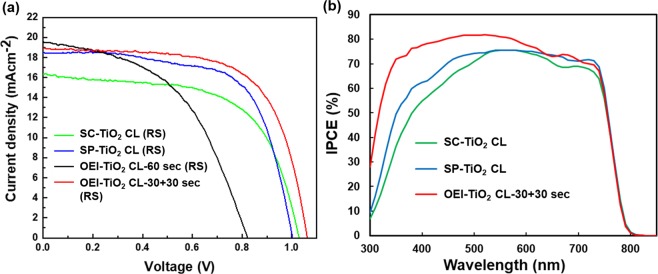
(**a**) RS *J–V* characteristics of PSCs made with SC-TiO_2_ CL, SP-TiO_2_ CL, OEI-TiO_2_ CL-60 sec, and OEI-TiO_2_ CL-30 + 30 sec. (**b**) IPCE spectra of PSCs made with SC-TiO_2_ CL, SP-TiO_2_ CL, and OEI-TiO_2_ CL-30 + 30 sec.

The photovoltaic performance of PSCs processed with SC-TiO_2_ CL, SP-TiO_2_ CL, OEI-TiO_2_ CL-60 sec, and OEI-TiO_2_ CL-30 + 30 sec is investigated and demonstrated. The *J–V* characteristics of the best-performing PSC devices are presented in Fig. 4a. The corresponding *J–V* parameters are summarized in Table 1. A comparison of the forward scan (FS) and reverse scan (RS) results are summarized in Table S1. Outline of the OEI-TiO_2_ CL based PSCs performance characteristics with different concentration of the TiO_2_ precursor solution (Table S2). The thickness of the OEI-TiO_2_ CL was tuned by changing (from 0.05 to 0.50 M) precursor solution concentration of the TiO_2_ and fabricated the resultant devices, as shown in Fig. S1. Ultimately, optimized and PSCs with 0.30 M based OEI-TiO_2_ CL-60 sec as the ETL exhibited PCEs as high as 7.86% compared to the varying concentration of TiO_2_ precursor solution based counterpart. Such poor device performance is caused by surface morphology cracks in the TiO_2_ CL that prevented efficient electron injection at the interface between TiO_2_ and perovskite, thus leading to a large leakage current and recombination of charge carriers^29^. On the other hand, to further improve the quality of OEI-TiO_2_ CL-60 sec and simultaneously improve the efficiency, the device was fabricated by OEI- TiO_2_ CL-30 + 30 sec spans. The resultant PSCs show a short-circuit current density (*J*_sc_) of 18.91 mA⋅cm^−2^, open-circuit voltage (*V*_oc_) of 1.06 V, fill factor (FF) of 0.66, and a PCE of 13.19%. In addition, the enhancement of *J*_sc_ and FF upon patterning the OEI-TiO_2_ CL for 30 sec film onto the OEI-TiO_2_ CL for 30 sec (referred to OEI-TiO_2_ CL-30 + 30 sec film) is attributed to the uniform, satisfactory surface coverage, and denser scaffold that has no visible cracks, thus facilitating more efficient carrier flow, as compared to that of the PSC made using the OEI- TiO_2_ CL-60 sec sample. In contrast, the OEI-TiO_2_ CL-30 + 30 sec-based PSCs exhibit PCEs as high as 13.19%, which is also better than that of PSCs formed using the SC-TiO_2_ CL, SP-TiO_2_ CL, and OEI-TiO_2_ CL-60 sec samples (10.27%, 12.19%, and 7.86%, respectively). The OEI-TiO_2_ CL-30 + 30 sec deposited-film strongly influences the TiO_2_ CL morphology, which significantly affects the resulting solar cell performance. However, the device characteristic *J–V* curves have hysteresis in all single-layer TiO_2_ CLs, which underestimates the actual performance of the PSCs (Table S1). In general, the hysteresis phenomenon impacted the rich number of both oxygen vacancies and electron traps on the surface of TiO_2_^30^. The incident photon-to-electron conversion efficiency (IPCE) is measured to further reveal the correctness of the output *J*_sc_, as shown in Fig. 4b. The IPCE coverage also significantly improved, especially in the range of ~380 nm to ~700 nm, with the OEI-TiO_2_ CL-30 + 30 sec spans compared to the counterparts. This significantly evidence the improved charge collection efficiency of OEI-TiO_2_ CL-30 + 30 sec-based perovskites, which could be solely related to high surface coverage (less shorting paths). The lower coverage for the devices made with SC-TiO_2_ CL and SP-TiO_2_ CL indicates that the perovskite quality and its interfacial connection with charge transport layers is poor.

**Table 1:** Outline of the parameters of PSCs with varying types of ETLs.

ETL		*J*_SC_ [mA⋅cm^−2^]	*V*_oc_ [V]	*FF*	PCE [%]
SC-TiO_2_ CL	Champion^a^	16.33	1.03	0.61	10.27
	Average ± SD	15.88 ± 1.07	0.90 ± 0.12	0.50 ± 0.10	7.24 ± 2.44
SP-TiO_2_ CL	Champion	18.49	1.00	0.65	12.19
	Average ± SD	17.76 ± 3.05	0.98 ± 0.15	0.55 ± 0.09	9.56 ± 2.48
OEI-TiO_2_ CL-60 sec	Champion	19.54	0.82	0.48	7.86
	Average ± SD	17.66 ± 1.46	0.70 ± 0.12	0.40 ± 0.03	4.99 ± 1.14
OEI-TiO_2_ CL-30 + 30 sec	Champion	18.91	1.06	0.66	13.19
	Average ± SD	18.04 ± 2.50	1.00 ± 0.06	0.61 ± 0.10	10.91 ± 2.06

Statistical analysis (average ± standard deviation) is based on the measurement of devices of each type. ^a^Champion refers to the device with the highest PCE.

**Figure 5:**
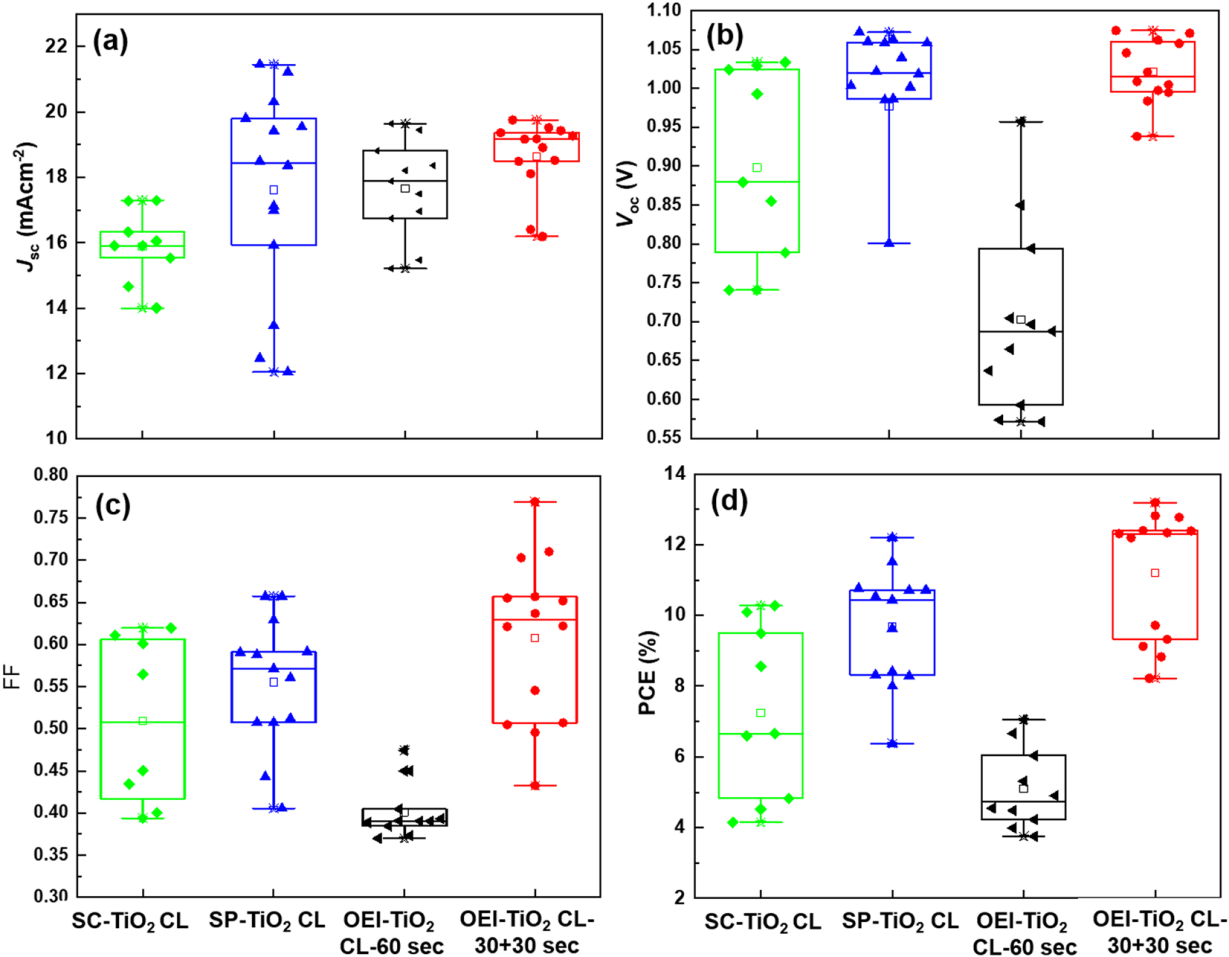
Average values of (**a**) *J*_sc_, (**b**) *V*_oc_, (**c**) FF, and (**d**) PCE obtained for SC-TiO_2_ CL, SP-TiO_2_ CL, OEI-TiO_2_ CL-60 sec, and OEI-TiO_2_ CL-30 + 30 sec-based PSCs.

Figure 5a–d show the statistical distribution of the four main parameters of the PSCs devices (*J*_sc_, *V*_oc_, FF, and PCE) as a function of the type of TiO_2_ CL as the ETL. To ensure the performance reproducibility of our results, we fabricated multiple PSCs with SC-TiO_2_ CL (9 devices), SP-TiO_2_ CL (15 devices), OEI-TiO_2_ CL-60 sec (11 devices), and OEI-TiO_2_ CL-30 + 30 sec (14 devices) as ETLs. The best control and highest reproducibility in terms of all the parameters are obtained from the uniform and full surface coverage OEI-deposited TiO_2_ CL-for 30 + 30 sec film. This implies that the OEI technique enables the control of the surface morphology and thickness of the TiO_2_ CL by simply modifying the coating time, which is crucial in inhibiting carrier recombination and enhancing the interface between the TiO_2_ CL and perovskite of the PSCs.

## Conclusions

For the first time, we applied a simple, novel, and promising OEI technique that does not require a vacuum environment to fabricate uniform TiO_2_ CL on FTO substrate; subsequently, we use the film as the ETL in planar PSCs. We tuned the surface morphology of the film OEI-TiO_2_ by simply changing the coating times to achieve a smooth and denser scaffold covering the entire substrate without visible cracks. The PSCs made with the optimized film as the ETL showed enhanced efficiency up to 13.19%. Therefore, OEI-deposited TiO_2_ CLs are candidates for use as ETLs in planar PSCs. Owing to further enhance the performance, the OEI-TiO_2_ CL-based PSCs, we suggest that surface engineering or elemental doping in the TiO_2_ CL will ensure a good interface between the TiO_2_ CL and perovskite, reducing the hysteresis.

In order to develop large-scale printing through OEI-approach, we will further apply multi-nozzle system as the single nozzle approach will be covered only 5 mm width. When each nozzle is set closely, OEI-approach will not be stable, and uniform printing will not be observed due to cross-talk of electric field around each nozzle. As the shield around each nozzle is set, the cross-talk will not be generated, and uniform and large-size printing will be achieved. However, by benchmarking our results, OEI-approach will be thoroughly explored to coat variety of substrates on the large scale for high-throughput large-area perovskite solar modules in our upcoming study. The rapid development toward scaling-up and large-area uniformity of OEI-substrates, coupled with low-cost fabricating capability that has a high material utilization rate and might reduce of production cost of resultant devices. This facile fabrication technique may be a new TiO_2_ CL ETL deposition candidate that helps to further improve the photovoltaic performance of planar small-scale and large- PSCs modules.

## Experimental

### Materials

FTO-patterned glass substrates were purchased from Asahi glass (Tokyo, Japan). Lead iodide (PbI_2_), methylammonium bromide (MABr), lead bromide (PbBr_2_), formamidinium iodide (FAI), and cesium iodide (CsI) were purchased from Tokyo Chemical Industry (Tokyo, Japan). The *N,N*-dimethylformamide (DMF; 99.5% purity) and dimethyl sulfoxide (DMSO; 99.5% purity) were purchased from Wako Pure Chemical (Osaka, Japan). Titanium diisopropoxide bis(acetylacetonate) (75 wt% in isopropanol) and 2-isopropanol (99.9% purity) were supplied from Sigma-Aldrich, Wako Pure Chemical, respectively.

### Fabrication of OEI-deposited TiO_2_ CL

The FTO-patterned glass substrates were cut into 25 mm squares and washed consecutively with a soap solution, distilled water, acetone, ethyl alcohol, and once more distilled water. Then, the UV ozone treatment was applied to FTO substrates for 15 min. The tip of a Terumo syringe (10 ml) was fitted with a San-ei Tech Ltd (Tokyo, Japan) TT taper nozzle (0.21 mm internal diameter), and the solution was filled up to 2.0 ml with the TiO_2_ solution. A clear yellow-orange precursor solution [from 0.05 to 0.50 M titanium diisopropoxide bis(acetylacetonate) in isopropanol], was filled into the ink tank. The optimized precursor solution of the OEI-TiO_2_ CL was 0.30 M. The nozzle was installed at the end of the tank. To maintain the electric field around the nozzle tip, a holed plate electrode (outside 100 × 145 mm^2^, hole diameter of 50 mm) was used between the nozzle tip and the FTO electrode. The FTO glass electrode was set on the XY linear stage that can be rotated. The gap between the FTO substrate and the tip of the nozzle was fixed at 60 mm, and the discharge time was 60 s. The applied voltage was 8.5 kV, the nozzle angle was 45°, and the substrate position was set in the vertical direction. The rotation speed was 2000 rpm. After depositing, the substrates were dried at 105 °C for 5 min, came after by sintering at 450 °C for 30 min in a muffle furnace and the resultant samples were used as ETLs. The OEI-deposited TiO_2_ CL surface coverage was evaluated for two different deposition conditions based on the duration of the discharge; samples made with 60 seconds and 30 + 30 seconds are referred to as OEI-TiO_2_ CL-60 and OEI-TiO_2_ CL-30 + 30, respectively. The OEI-TiO_2_ CL-30 + 30 sample preparation procedure as follows. First, TiO_2_ precursor solution was patterned by OEI for 30 seconds as usual. Then, the as-deposited substrate was dried at 105 °C for 5 min and allowed to cool down to room temperature. After that, the TiO_2_ precursor solution was further deposited for 30 seconds onto the existing film. After the deposition, the substrates were dried at 105 °C for 5 min, followed by sintering at 450 °C for 30 min. Again, the samples were cooled slowly to room temperature and subsequently used as ETLs.

### Fabrication of PSCs

The perovskite precursor solution with Cs 5% was made by combining with FA/MA in DMF and DMSO with the ratio of 4:1 mixed solvents. The descriptive solution preparation and spin-coating process is described elsewhere^21^. The FAI (1 M), PbI_2_ (1.1 M), MABr (0.2 M), and PbBr_2_ (0.2 M) were mixed in anhydrous DMF: DMSO (4:1). Since then, the Cs 5% solution was added in the perovskite precursor solution to achieve the Cs based perovskite precursor solution. As deposited samples were annealed at 100 °C for 60 min. For the hole-transport layer (HTL), the detailed precursor preparation is described elsewhere^21^. At last, vacuum deposited Au (100 nm) was formed as an electrode on the HTL to complete the device.

### Characterization

Field emission scanning electron microscopy (S-4800, Hitachi High-Tech, Tokyo, Japan) was used to examine the surface morphology. The current density *versus* voltage (*J–V*) curves were measured and analyzed with the simulated solar conditions (100 mW⋅cm^−2^, AM 1.5, 1 sun intensity) utilizing a Keithley 2401 digital source meter. A monochromatic xenon arc light scheme (Bunkoukeiki, SMI-250JA) was used to measure the IPCE of the resultant devices. The 0.09 cm^2^ was the active area of the fabricated devices.

## Supplementary information

Supporting Information
