# Supplementary material for: Oblique Electrostatic Inkjet-Deposited TiO2 Electron Transport Layers for Efficient Planar Perovskite Solar Cells
Source: Sci Rep. 2019 Dec 20;9:19494. doi: 10.1038/s41598-019-56164-w (PMC6925098; doi:10.1038/s41598-019-56164-w)
Supplement: Supplementary file 1 — Supporting Information [file 41598_2019_56164_MOESM1_ESM.pdf]

Supporting Information

Oblique Electrostatic Inkjet-Deposited TiO_2 Electron Transport Layers for Efficient Planar Perovskite Solar Cells

Md. Shahiduzzaman[1,2,*], Toshiharu Sakuma[4], Tetsuya Kaneko[4], Koji Tomita[3], Masao Isomura[4], Tetsuya Taima[1], Shinjiro Umezu[5,*], Satoru Iwamori[2,4,*]

[1]*Nanomaterials Research Institute (NanoMaRi), Kanazawa University, Kakuma, Kanazawa 920-1192, Japan*

[2]*Research Institute of Science and Technology (RIST), Tokai University, Kitakaname, Hiratsuka 259-1292, Japan*

[3]*Department of Chemistry, School of Science, Tokai University, Kitakaname, Hiratsuka 259-1292, Japan*

[4]*Graduate School of Engineering, Tokai University, Kitakaname, Hiratsuka 259-1292, Japan*

[5]*Department of Modern Mechanical Engineering, Waseda University, 3-4-1 Ookubo, Shinjyuku-ku, Tokyo, 269-8555 Japan*

*Corresponding author(s): shahiduzzaman@se.kanazawa-u.ac.jp (M. Shahiduzzaman); umeshin@waseda.jp (S. Umezu); iwamori@tokai-u.jp (S. Iwamori),

Tel/Fax: +81-76-264-4937

Table of Contents

Figure S1. Reverse scan *J–V* characteristics of OEI-TiO_2 CL based PSCs fabricated with 0.05, 0.10, 0.15, 0.20, 0.30, 0.40, and 0.50 M precursor solution of TiO_2……….S3

Table S1. Summary of PSCs performance characteristics with different TiO_2 CL as the ETLs…………………………………………………………………………………...S4

Table S2. Outline of the OEI-TiO_2 CL based PSCs performance characteristics with different concentration of the TiO_2 precursor solution of 0.05, 0.10, 0.15, 0.20, 0.30, 0.40, and 0.50 M…………………………………………………………………….S5

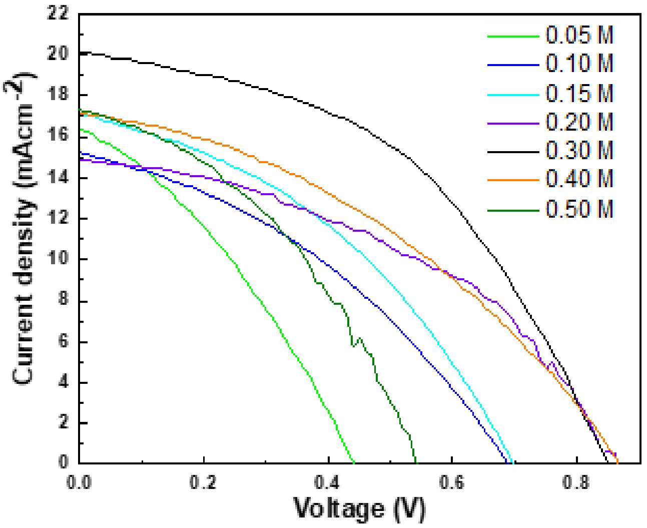

Figure S1. Reverse scan *J–V* characteristics of OEI-TiO_2 CL based PSCs fabricated with 0.05, 0.10, 0.15, 0.20, 0.30, 0.40, and 0.50 M precursor solution of TiO_2.

Table S1. Summary of PSCs performance characteristics with different TiO_2 CL as the ETLs.

Compact layer	Scan direction	J_{sc} (mA cm^{-2})	V_{oc} (V)	FF	PCE (%)
SC-TiO_2 CL	Reverse	16.33	1.03	0.61	10.27
	Forward	17.99	1.01	0.46	8.32
SP-TiO_2 CL	Reverse	18.49	1.00	0.66	12.19
	Forward	19.17	1.00	0.58	11.14
OEI-TiO_2 CL-60 sec	Reverse	19.54	0.82	0.48	7.86
	Forward	18.80	0.70	0.36	4.90
OEI-TiO_2 CL-30+30 sec	Reverse	18.91	1.06	0.66	13.19
	Forward	20.19	1.03	0.57	11.82

Table S2. Outline of the OEI-TiO_2 CL based PSCs performance characteristics with different concentration of the TiO_2 precursor solution of 0.05, 0.10, 0.15, 0.20, 0.30, 0.40, and 0.50 M.

**Concentration of the TiO_2 precursor solution**	**Scan direction**	J_{sc} **(mA cm^{-2})**	V_{oc} **(V)**	**FF**	**PCE (%)**
0.05 M	Reverse	16.37	0.44	0.33	2.45
	Forward	16.52	0.49	0.30	2.51
0.10 M	Reverse	15.25	0.68	0.37	3.89
	Forward	17.82	0.85	0.40	6.18
0.15 M	Reverse	13.40	0.38	0.34	1.75
	Forward	17.19	0.69	0.39	4.69
0.20 M	Reverse	14.90	0.85	0.43	5.58
	Forward	18.25	0.94	0.41	7.16
0.30 M	Reverse	19.54	0.82	0.48	7.86
	Forward	18.80	0.70	0.36	4.90
0.40 M	Reverse	17.19	0.86	0.38	5.70
	Forward	16.98	0.73	0.41	5.10
0.50 M	Reverse	17.34	0.54	0.39	3.72
	Forward	19.37	0.66	0.43	5.68
